# AFsample: improving multimer prediction with AlphaFold using massive sampling

**DOI:** 10.1093/bioinformatics/btad573

**Published:** 2023-09-15

**Authors:** Björn Wallner

**Affiliations:** Division of Bioinformatics, Department of Physics, Chemistry and Biology, Linköping University, SE-581 83 Linköping, Sweden

## Abstract

**Summary:**

The AlphaFold2 neural network model has revolutionized structural biology with unprecedented performance. We demonstrate that by stochastically perturbing the neural network by enabling dropout at inference combined with massive sampling, it is possible to improve the quality of the generated models. We generated ∼6000 models per target compared with 25 default for AlphaFold-Multimer, with v1 and v2 multimer network models, with and without templates, and increased the number of recycles within the network. The method was benchmarked in CASP15, and compared with AlphaFold-Multimer v2 it improved the average DockQ from 0.41 to 0.55 using identical input and was ranked at the very top in the protein assembly category when compared with all other groups participating in CASP15. The simplicity of the method should facilitate the adaptation by the field, and the method should be useful for anyone interested in modeling multimeric structures, alternate conformations, or flexible structures.

**Availability and implementation:**

AFsample is available online at http://wallnerlab.org/AFsample.

## 1 Introduction

The unprecedented accuracy of AlphaFold version 2 (AF2) (Jumper *et al.* 2021) has transformed the field of computational and structural biology. It is now possible to achieve highly accurate predictions on par with experimentally determined structures. AF2 has rapidly become the go-to method for protein structure prediction for both monomers and multimer prediction pipelines (Cramer 2021).

A key to the success of AF2 is its ability to assess the accuracy of its own predictions. AF2 estimates the per-residue accuracy using the predicted LDDT (pLDDT; Mariani *et al.* 2013). In addition, it also calculates a predicted TMscore (pTM; Zhang and Skolnick 2004) from the predicted aligned error (PAE) matrix (Jumper *et al.* 2021). The correlation for pLDDT and pTM to its actual values are 0.76 and 0.85, respectively (Jumper *et al.* 2021), and this correlation is maintained even for high-quality predictions. For multimer prediction, an interchain predicted TMscore (ipTM) is calculated from the PAE of the interchain distances (Jumper *et al.* 2021).

Given enough evolutionary-related sequences, AF2 predicts monomers with very high accuracy even without using structural templates (Jumper *et al.* 2021). However, for multimers, this is not necessarily true (Bryant *et al.* 2022), the evolutionary signal constraining multimers is much weaker, and for these cases, more sampling might improve the prediction. The need for more sampling was also the reason why the default number of sampled models in AlphaFold-Multimer v1 was increased from 5 to 25 in AlphaFold-Multimer v2. Furthermore, predicting transient interactions or interactions with flexible binding partners, such as short peptides or disordered regions, requires even more sampling to achieve optimal performance (Johansson-Åkhe and Wallner 2022).

For complex cases, simply increasing the number of sampled models might not be enough if the evolutionary constraints have trapped the prediction in a local minimum in the conformational landscape (Roney and Ovchinnikov 2022) or the if the evolutionary constraints are weak. In such cases, increasing the number of times the prediction is recycled in the network can improve performance (Mirdita *et al.* 2022). Another option is to randomly perturb or alter the input multiple sequence alignment (MSA), which has been shown to enable better sampling of the conformational landscape and prediction of multiple conformational states (Wayment-Steele *et al.* 2022).

An alternative way to achieve more diversity among the generated models is to enable the dropout layers in the neural network (Johansson-Åkhe and Wallner 2022, Mirdita *et al.* 2022). The dropout layers in a neural network are commonly used only when training the networks to cause them to learn different redundant solutions to the same problem by stochastically dropping some of their weights (putting them to zero). The dropout rate in the AlphaFold2 network is 10%–25%, depending on the network module. By activating these layers at inference, the network will naturally sample the uncertainties (Gal and Ghahramani 2016) in the structure prediction, and the structural diversity of the sampled models will be increased. Maintaining the same dropout rates as during training makes sense because the model has been designed to function effectively under these conditions. However, there may be instances where increasing the dropout rates could prove beneficial to increase the diversity and this is something we like to follow up on in future studies.

## 2 Materials and methods

### 2.1 Databases

Sequence databases were downloaded on 22 April 2022, and the PDB was updated 2 May 2022, using the download scripts provided by DeepMind (https://github.com/deepmind/alphafold/). The following versions were used:

- Uniclust30 (Mirdita *et al.* 2017) version: UniRef30_2021_03.
- Uniref90 (Suzek *et al.* 2015) from 22 April 2022.
- Uniprot, TrEMBL, SwissProt, from 22 April 2022.
- BFD database (Steinegger and Söding 2018).
* .ffindex MD5: 26d48869efdb50d036e2fb9056a0ae9d.
- Mgnify version: 2018_12.
- PDB from 2 May 2022.

### 2.2 Benchmark

In the CASP15 benchmark, AFsample (Wallner) and AlphaFold-Multimer baseline (NBIS-AF2-multimer) were run with exactly the same MSAs and templates. The alignments were created with the large database setting: —db_preset=full_dbs using the AlphaFold-Multimer baseline server (NBIS-AF2-multimer). They were made available by the CASP organizers, and these were the MSAs used by the Wallner group in CASP15. The DockQ (Basu and Wallner 2016) scores for all methods that participated in CASP15 were downloaded from the CASP15 website. In the case of multiple interfaces, DockQ is calculated for each interface and then averaged. The rank 1 models from each method were used to calculate the average DockQ for the multimer targets for each method.

## 3 Results

**Figure 1.**
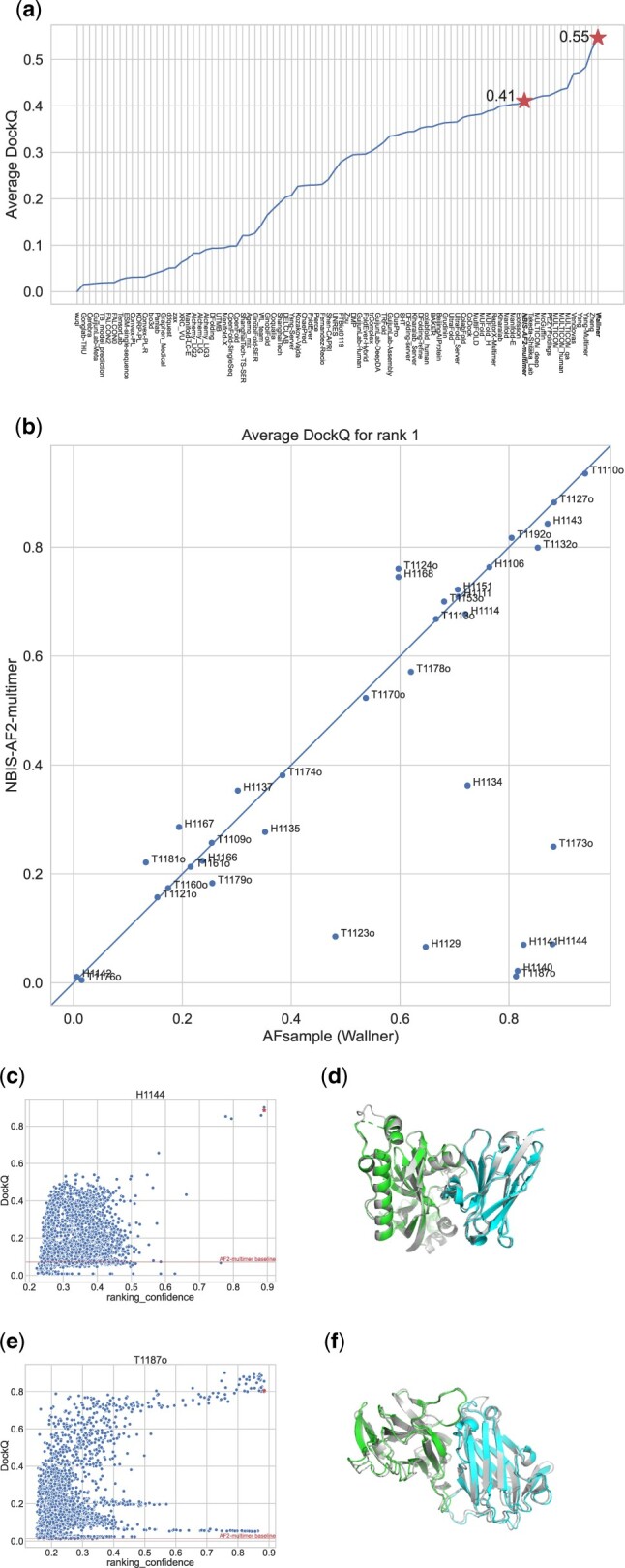
AFsample (Wallner) performance on common CASP15 multimer target compared AlphaFold-Multimer v2 baseline (NBIS-AF2-multimer). (a) Average DockQ on common multimeric targets for all groups in CASP15. The lower star represents the AlphaFold-Multimer v2 baseline (NBIS-AF2-multimer), and the upper star represents AFsample (Wallner). (b) DockQ comparison to AlphaFold-Multimer v2 baseline per CASP15 target. (c) Ranking_confidence score versus DockQ for models sampled for CASP target H1144. (d) CASP target H1144, native chain A (green, left) and B (cyan, right), rank 1 prediction in grey, DockQ = 0.88. (e) Ranking_confidence score versus DockQ for models sampled for CASP target T1187. (f) CASP target T1187o, native chain A (green) and B (cyan), rank 1 prediction in grey, DockQ = 0.81.

Here, we present AFsample that significantly improves over AlphaFold-Multimer v2 baseline (NIBS-AF2-Multimer). The method was the most successful in CASP15 for multimer prediction (Wallner 2023) (Fig. 1a). It improved the average DockQ (Basu and Wallner 2016) from 0.41 for AF2-Multimer (NBIS-AF2-multimer) to 0.55, with eight targets showing considerable improvements with >0.4 DockQ units, essentially going from incorrect to high-quality predictions (Fig. 1b).

In short, AFsample generates a large pool of models using different settings (Supplementary Table S1). The self-assessment ranking score (ranking_confidence), which for multimer is a linear combination of the predicted interface TMscore (ipTM) and the predicted TMscore (pTM), 0.8ipTM+0.2pTM, is used for selection. Six different settings were used, and they all involved using dropout to increase the model diversity. Both v1 and v2 of the multimer neural network weights and increased number of recycles were also utilized to increase the diversity further. The number of recycles for v1 and v2 were optimized in a previous study of peptide–protein predictions (Johansson-Åkhe and Wallner 2022). Four settings involved selective dropout, where we only activated dropout in the Evoformer part of the network and not in the structural module. Despite the name, the bulk of structure prediction in AF2 is not performed in the structural module but in the much more extensive Evoformer network. In a previous study, we observed an improved correlation between ranking_confidence and actual DockQ using selective dropout with no dropout in the structural module (Johansson-Åkhe and Wallner 2022). For each setting, on the order of 1000 models were generated per setting for a total of 6000 models per target.

It is clear the sole reason for the improved performance of AFsample is improved sampling as AFsample was using identical MSAs as the AlphaFold-Multimer v2 baseline. The need for sampling is illustrated further by target H1144 (Fig. 1c and d). Target H1144 is a nanobody interaction, nanobodies and regular antibody interactions are particularly challenging to model since the interaction with the antigen is highly specific, and the exact shape and location of the epitopes can vary significantly depending on the antigen (Chiu *et al.* 2019). Here, only 3 of the 6000 models obtained a ranking_confidence > 0.8, of which all were of high quality with a DockQ > 0.8. In fact, only five high-quality models could be found in the whole set of 6000 models sampled. Thus, without substantial sampling, most likely, no high-quality model would have been generated at all.

The other example is T1187o (Fig. 1e and f). Target T1187o is a dimer of the Uniprot ID Q94EW1. The AlphaFold prediction of the monomer is almost perfect (https://alphafold.ebi.ac.uk/entry/Q94EW1), but the model of the dimer is completely wrong with a DockQ very close to zero (Fig. 1e). However, with improved sampling, it is possible to generate several high-quality models (38/6000). Still, selecting the best possible model was not straightforward as one-third of the models with a ranking_confidence > 0.8 actually had a wrong domain orientation indicated by the low DockQ score.

Since the development of AFsample, AlphaFold-Multimer v3 has been released and the AFsample code has been updated to use v3 as well as v1 and v2 when generating models. For the examples above, v3 generates models with DockQ 0.44 and 0.09 for H1144 and T1187o, respectively, both are better than v2, but not as good as AFsample. However, it is important to note that the v3 predictions are not blind since it was released after CASP15 and several targets were already available. Still, in general it should not be a disadvantage to include an additional set of weights to increase the diversity and the likelihood of generating high-quality models with high confidence scores.

AFsample requires more computational time than AF2, as it generates 240× models, and including the extra recycles, the overall timing is ∼1000× more costly than the baseline. Thus, a good strategy is first to run the AF2 baseline prediction and only use more sampling if needed. A good criterion for this is to use a ranking_confidence > 0.8 and require at least a couple of structures at this level of confidence. Finally, given that AlphaFold inference in many cases is relatively fast, a minute or less per model, a couple of days on a single GPU to get high-quality results for a difficult target is not an enormous amount of computational time.

The results from CASP15 demonstrate that the best way to model multimeric protein assemblies today is to use AFsample. However, it is very likely that sampling will improve the performance of AlphaFold in any setting. Dynamics, flexibility, or simply the sheer complexity of large molecular assemblies will all require more sampling. The success of sampling relies heavily on the excellent internal scoring function in AlphaFold, which so far has proven to be exceptionally good.

## Supplementary Material

btad573_Supplementary_Data

## References

[btad573-B1] Basu S , Wallner B. DockQ: a quality measure for protein-protein docking models. PLoS One 2016;11:e0161879. 27560519 10.1371/journal.pone.0161879 PMC4999177

[btad573-B2] Bryant P , Pozzati G, Elofsson A. Improved prediction of protein-protein interactions using AlphaFold2. Nat Commun 2022;13:1265. 35273146 10.1038/s41467-022-28865-w PMC8913741

[btad573-B3] Chiu ML , Goulet DR, Teplyakov A et al Antibody structure and function: the basis for engineering therapeutics. Antibodies 2019;8:55. 31816964 10.3390/antib8040055 PMC6963682

[btad573-B4] Cramer P. AlphaFold2 and the future of structural biology. Nat Struct Mol Biol 2021;28:704–5. 34376855 10.1038/s41594-021-00650-1

[btad573-B5] Gal Y , Ghahramani Z. Dropout as a Bayesian approximation: representing model uncertainty in deep learning. PMLR 2016;48:1050–1059.

[btad573-B6] Johansson-Åkhe I , Wallner B. Improving peptide-protein docking with AlphaFold-Multimer using forced sampling. Front Bioinform 2022;2:959160. 36304330 10.3389/fbinf.2022.959160 PMC9580857

[btad573-B7] Jumper J , Evans R, Pritzel A et al Highly accurate protein structure prediction with AlphaFold. Nature, 2021;596:583–9. 34265844 10.1038/s41586-021-03819-2 PMC8371605

[btad573-B8] Mariani V , Biasini M, Barbato A et al LDDT: a local superposition-free score for comparing protein structures and models using distance difference tests. Bioinformatics 2013;29:2722–8. 23986568 10.1093/bioinformatics/btt473 PMC3799472

[btad573-B9] Mirdita M , Schütze K, Moriwaki Y et al ColabFold: making protein folding accessible to all. Nat Methods 2022;19:679–82. 35637307 10.1038/s41592-022-01488-1 PMC9184281

[btad573-B10] Mirdita M , von den Driesch L, Galiez C et al Uniclust databases of clustered and deeply annotated protein sequences and alignments. Nucleic Acids Res 2017;45:D170–6. 27899574 10.1093/nar/gkw1081 PMC5614098

[btad573-B11] Roney JP , Ovchinnikov S. State-of-the-art estimation of protein model accuracy using AlphaFold. Phys Rev Lett 2022;129:238101. 36563190 10.1103/PhysRevLett.129.238101 PMC12178128

[btad573-B12] Steinegger M , Söding J. Clustering huge protein sequence sets in linear time. Nat Commun 2018;9:2542. 29959318 10.1038/s41467-018-04964-5 PMC6026198

[btad573-B13] Suzek BE , Wang Y, Huang H, et al; The UniProt Consortium. UniRef clusters: a comprehensive and scalable alternative for improving sequence similarity searches. Bioinformatics 2015;31:926–32. 25398609 10.1093/bioinformatics/btu739 PMC4375400

[btad573-B14] Wallner B. Improved multimer prediction using massive sampling with AlphaFold in CASP15. Proteins 2023;1-13. doi:10.1002/prot.26562. 37548092

[btad573-B15] Wayment-Steele HK , Ovchinnikov S, Colwell L et al Prediction of multiple conformational states by combining sequence clustering with AlphaFold2. bioRxiv, https://doi.org/2022.10.17.512570.

[btad573-B16] Zhang Y , Skolnick J. Scoring function for automated assessment of protein structure template quality. Proteins 2004;57:702–10. 15476259 10.1002/prot.20264

