# Supplementary material for: AFsample: improving multimer prediction with AlphaFold using massive sampling
Source: Bioinformatics. 2023 Sep 15;39(9):btad573. doi: 10.1093/bioinformatics/btad573 (PMC10534052; doi:10.1093/bioinformatics/btad573)
Supplement: btad573_Supplementary_Data [file btad573_supplementary_data.pdf]

Bioinformatics
doi.10.1093/bioinformatics/xxxxxx
Advance Access Publication Date: Day Month Year
Application Note

Structural Bioinformatics

Supplementary information AFsample: Improving Multimer Prediction with AlphaFold using Aggressive Sampling

Björn Wallner[1,*]

[1] Division of Bioinformatics, Department of Physics, Chemistry and Biology, Linköping University, SE-581 83 Linköping, Sweden

*To whom correspondence should be addressed.

1 Extended Methods

Version	Templates	Dropout	Recycles
multimer_v1	Yes	Yes	3
multimer_v1	No	Yes*	3
multimer_v1	No	Yes*	21
multimer_v2	Yes	Yes	3
multimer_v2	No	Yes*	3
multimer_v2	No	Yes*	9

Table 1. Different settings of AlphaFold used in AFsample, Version refers to the version of the multimer neural network weights, Templates refers to if structural templates were used or not, Dropout refers to if dropout was enabled, Recycles refers to how many recycles was used (default 3).
*No dropout in structural module

AFsample is available as a command line interface using a modified version of the official AlphaFold release. The modified version is streamlined to produce many models, contains functionality to parallelize the computations to independent jobs, and exposes several internal parameters of AlphaFold to allow using it at its full potential. A script `run_afsample.sh` is provided that reproduce the method that participated in CASP15 under the name `Wallner`.

Filter the output. To enable the generation of thousands of models, the amount of data saved per model was limited to the predicted aligned error matrix (`PAE`), per residues predicted LDDT (`pLDDT`), overall predicted TMscore(`pTM`), predicted interchain TMscore (`ipTM`), and `ranking_confidence`. This reduces the size of the data saved per model by approximately a factor 100. The flag `--output_all_results` will restore the default behavior and output all data structures.

Checkpointing. Checkpointing is made default, it will not recalculate MSAs if they exists and if a model exists it will continue to the next model.

Added functionality. The most important modifications and exposed functionalities are described below:

`--model_preset` Modified to allow using both v1 and v2 models, the following presets are allowed: `multimer_v1`, `multimer_v2`, `multimer` (defaults to v2), `multimer_all` (both v1 and v2)
`--nstruct` the number of structures to output, works both for monomer and multimer protocols and replaces the `--num_multimer_predictions_per_model` which was exclusive to the multimer protocol.
`--max_recycles` the number of times a prediction is recycled in the neural network, default is 3.
`--dropout` enables the dropout layers at inference.
`--dropout_structure_module` if `False` it will not have the dropout layers enabled in the structure module.
`--suffix` option to add a descriptive suffix to each model name to enable output to the same output folder.
`--no_templates` do not use any templates, faster than filter by date which requires parsing all hits.
`--seq_only` will only run the sequence searches to create the MSAs and template hits, useful to prepare input files for larger runs.
`--input_msa` option to input a multiple sequence alignment in STOCKHOLM format.
`--nstruct_start` which structure to start with, useful to split a large job in many smaller by using `--nstruct_start 20` and `--nstruct 21` it will create models `20` and `21`.
`--models_to_use` option to specify which neural network models from `model_preset` to use. i.e `model_1_multimer_v2, model_3_multimer_v2` will only run model 1 and model 3 from `multimer_v2`.

Databases. Sequence databases were downloaded on April 22, 2022, and the PDB was updated May 2, 2022, using the download scripts provided by DeepMind (`https://github.com/deepmind/alphafold/`). The following version were used:

- Uniclust30 (Mirdita *et al.*, 2017) version: `UniRef30_2021_03`
- Uniref90 (Suzek *et al.*, 2015) from April 22, 2022.
- Uniprot, TrEMBL+SwissProt, from April 22, 2022.
- BFD database (Steinegger and Söding, 2018) `bfd_metaclust_clu_complete_id30_c90_final_seq.sorted_opt_cs219.ffindex` `MD5 hash: 26d48869efdb50d036e2fb9056a0ae9d`
- Mgnify version: `2018_12`
- PDB from May 2, 2022.

All-atom relaxation. In regular AlphaFold each model is constrained relaxed in the Amber99sb force field (Hornak *et al.*, 2006) using

© The Author 2023. Published by Oxford University Press. All rights reserved. For permissions, please e-mail: journals.permissions@oup.com

openMM (Eastman *et al.*, 2017). To save computational time the all-atom relaxation step is skipped for each model. Instead a script `run_relax_from_results_pkl.py` is provided, that performs the relaxation step for a given result `pickle`. Since none of the scores depend on this step, the relaxation can be performed only for a smaller subset of the models that are high scoring or are selected by some other criteria.

Benchmark.In the CASP15 benchmark both AFsample (`Wallner`) and AF2-multimer baseline was run with exactly the same multiple sequence alignments (MSAs) and templates. The alignments were created with the large database setting: `--db_preset=full_dbs` using the AF2-multimer baseline server. They were made available by the CASP organisers, and these were the MSAs used by the `Wallner` group in CASP15. The DockQ (Basu and Wallner, 2016) scores for all methods that participated in CASP15 were downloaded from the CASP15 website. In the case of multiple interfaces, DockQ is calculated for each interface and then averaged. The rank 1 models from each method were used to calculate the average DockQ for the multimer targets for each method.

Data Availability

The MSAs and template information used in the CASP15 benchmark is available here: `http://bioinfo.ifm.liu.se/casp15/`

Code Availability

AFsample is free, open-source software (Apache) and is available from here: `http://wallnerlab.org/AFsample`

References

Basu, S. and Wallner, B. (2016). DockQ: a quality measure for protein-protein docking models. *PloS one*, **11**(8), e0161879.

Eastman, P., Swails, J., Chodera, J. D., McGibbon, R. T., Zhao, Y., Beauchamp, K. A., Wang, L.-P., Simmonett, A. C., Harrigan, M. P., Stern, C. D., Wiewiora, R. P., Brooks, B. R., and Pande, V. S. (2017). OpenMM 7: Rapid development of high performance algorithms for molecular dynamics. *PLoS Computational Biology*, **13**(7), e1005659.

Hornak, V., Abel, R., Okur, A., Strockbine, B., Roitberg, A., and Simmerling, C. (2006). Comparison of multiple Amber force fields and development of improved protein backbone parameters. *Proteins: Structure, Function, and Bioinformatics*, **65**(3), 712–725.

Mirdita, M., von den Driesch, L., Galiez, C., Martin, M. J., Söding, J., and Steinegger, M. (2017). Uniclust databases of clustered and deeply annotated protein sequences and alignments. *Nucleic Acids Research*, **45**(D1), D170–D176.

Steinegger, M. and Söding, J. (2018). Clustering huge protein sequence sets in linear time. *Nature Communications*, **9**(1), 2542.

Suzek, B. E., Wang, Y., Huang, H., McGarvey, P. B., Wu, C. H., and Consortium, U. (2015). UniRef clusters: a comprehensive and scalable alternative for improving sequence similarity searches. *Bioinformatics*, **31**(6), 926–932.
